# Synthesis of porous polymer/tissue paper hybrid membranes for switchable oil/water separation

**DOI:** 10.1038/s41598-017-03265-z

**Published:** 2017-06-08

**Authors:** Cong-Xiao Cao, Jiayin Yuan, Jin-Pei Cheng, Bao-Hang Han

**Affiliations:** 1 State Key Laboratory of Elemento-organic Chemistry, Collaborative Innovation Center of Chemical Science and Engineering (Tianjin), College of Chemistry, Nankai University, Tianjin, 300071 China; 2 CAS Key Laboratory of Nanosystem and Hierarchical Fabrication, CAS Center for Excellence in Nanoscience, National Center for Nanoscience and Technology, Beijing, 100190 China; 3 Department of Colloid Chemistry, Max Planck Institute of Colloids and Interfaces, Potsdam, D-14424 Germany; 4 Department of Chemistry and Biomolecular Science & Center for Advanced Materials Processing, Clarkson University, Potsdam, New York 13699-5814 USA

## Abstract

The unusually broad physical and chemical property window of ionic liquids allows for a wide range of applications, which gives rise to the recent spring-up of ionic liquid-based functional materials. *Via* solvothermal copolymerization of a monomeric ionic liquid and divinylbenzene in the presence of a tissue paper in autoclave, we fabricated a flexible porous polymer/paper hybrid membrane. The surface areas of the hybrid membranes depend on the weight fraction of the copolymer impregnated inside the tissue paper. The as-prepared hybrid membrane shows controlled surface wettability in terms of ethanol wetting and ethanol removal by harsh drying condition. This unique property provides the hybrid membrane with switchable oil/water separation function, thus of practical values for real life application.

## Introduction

Oil/water separation techniques have attracted much interest to address the environmental and ecological concerns caused by the leakage of oil and industrial organic pollutants into aqueous surroundings^1–4^. Following the recent advance in colloid and interface science, interfacial materials have been widely examined and used for efficient and recyclable oil/water separation^5–8^. Wettability determined by surface topography and surface chemical composition is important to interfacial materials^9–11^. The synergistic effect between surface topography and surface chemical composition is a key to vary the wettability towards either oil or water and dominates their selective separation^12^. Therefore reversible switching of a surface state between being hydrophilic and hydrophobic is crucial, which can be achieved by engineering stimuli-responsive surfaces with well-designed nano- or microstructures. These external stimuli include electrical potential^13, 14^, temperature^15, 16^, light illumination^17, 18^, pH value^19, 20^, gas^21^, and solvent^22–25^. Using solvent to control the surface hydrophilicity/hydrophobicity is a simple and practical approach. For example, a thin polymer coating built up from two polymer brushes of distinctively different hydrophilicity/hydrophobicity exhibits solvent-determined topmost surface components. The hydrophilic polymer chains collapsed in nonpolar solvents, while the hydrophobic ones extended and became the topmost surface layer. In contrast, the hydrophilic chains dominated the surface property in polar solvents^26^. This control allows solvents as external stimulus to modulate the wettability of a membrane surface. Generally speaking, responsive polymer materials for controllable oil/water separation are of great significance. In spite of recent advance and progress in this field^21, 26^, there remains much room for materials scientists to develop and improve the state-of-the-art techniques, *e.g*. by building up more simple setups and reducing synthetic complexity.

Meanwhile, ionic liquids have been particularly attractive recently because of their broad applications in the fields of chemical synthesis and materials science, including catalysis^27, 28^, electrochemistry^29^, green chemistry and clean energy^30–33^, functional nanomaterials^34–36^, *etc*. Their unique characteristics of facile tuneability of physical properties are useful in wettability control^37^. There have been reports on ionic liquids’ wettability including electro-wetting of ionic liquids used as probe fluids and super-wettability^38–40^. Currently, the study on ionic liquids has extended to their polymers, *i.e*. poly(ionic liquid)s (PILs), which are prepared from polymerization of ionic liquids, to broaden their materials applications^41–47^. PILs can combine the unique characters of ionic liquids with the common features of polymers^48, 49^. This marriage has created an alternative material class that is able to switch surfaces between being hydrophobic and hydrophilic in a different mechanism. For example, the wettability of PILs could be altered easily by exchanging appropriate counterions, either cations or anions, without modifying the polymer backbone^50–53^.

Here, *via* solvothermal copolymerization of an ionic liquid and divinylbenzene using tissue paper as template, we prepared a porous polymer-paper hybrid membrane bearing high porosity and mechanical flexibility. The wettability of the membrane changed repeatedly by solvent wetting and solvent removal at elevated temperature under vacuum, which was demonstrated to be useful in switchable oil/water separation.

## Results

### Fabrication of porous polymer-paper hybrid membranes (PPHMs)

**Figure 1:**
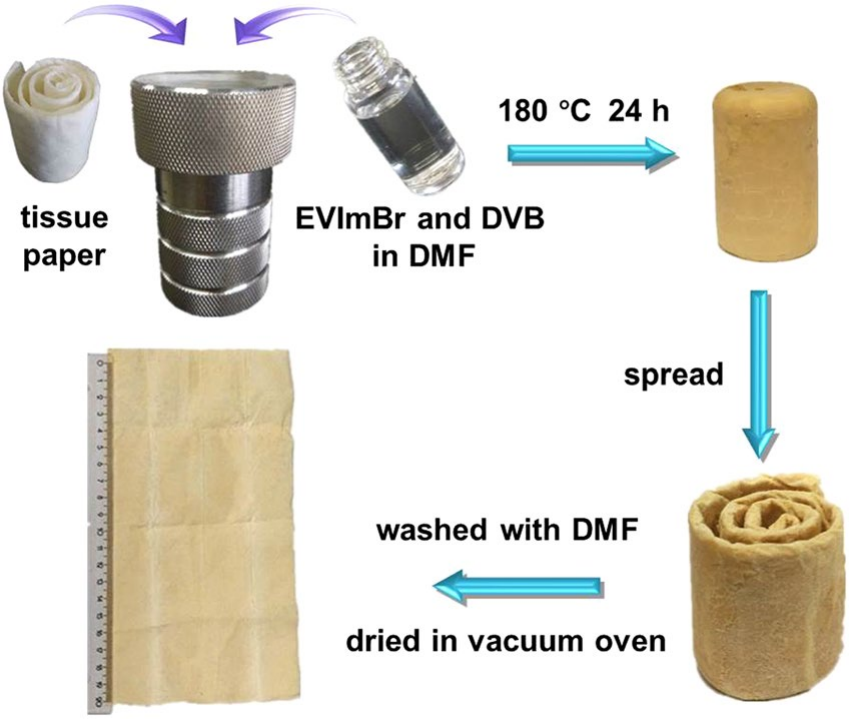
The schematic illustration of the preparation of PPHMs *via* solvothermal copolymerization of EVImBr and DVB in DMF in the presence of tissue paper in an autoclave.

Our approach features in general easy experimental implementation. Here, a one-step facile and scalable process (Fig. 1) to prepare PPHMs was developed *via* using cellulose microfiber-based tissue papers as macroporous template. In such tissue paper, the micron-sized interstice that is built up by random stacking of cellulose microfibers is used here to accommodate the microporous poly(EVImBr-*co*-DVB) copolymer network. In detail, the PPHM was tactfully prepared through solvothermal copolymerization of an ionic liquid monomer 1-ethyl-3-vinylimidazolium bromide (EVImBr) and divinylbenzene (DVB) in the presence of tissue papers in autoclave. Experimentally, a mixture solution of EVImBr and DVB in DMF was loaded into a Teflon-sealed autoclave, in which a densely packed roll of tissue paper was added and fully wetted by the liquid mixture. The solvothermal treatment at 180 °C for 24 h produced a poly(EVImBr-*co*-DVB) copolymer in the autoclave. DMF as solvent as well as the molecular template (porogen) was added here in an appropriate volume fraction so a pale yellow monolith was obtained after the polymerization. Solvothermal conditions were found necessary for the preparation of porous polymer-paper hybrid membranes. After cooling down, the monolith was crushed to release the tissue paper roll impregnated by the porous copolymer network as the final product. Here a series of hybrid membranes were synthesized with molar ratios of EVImBr and DVB varying from 0 (PPHM-0), 0.05 (PPHM-1), 0.1 (PPHM-2), 0.2 (PPHM-3), to 0.3 (PPHM-4) (Supplementary Table S1).

**Figure 2:**
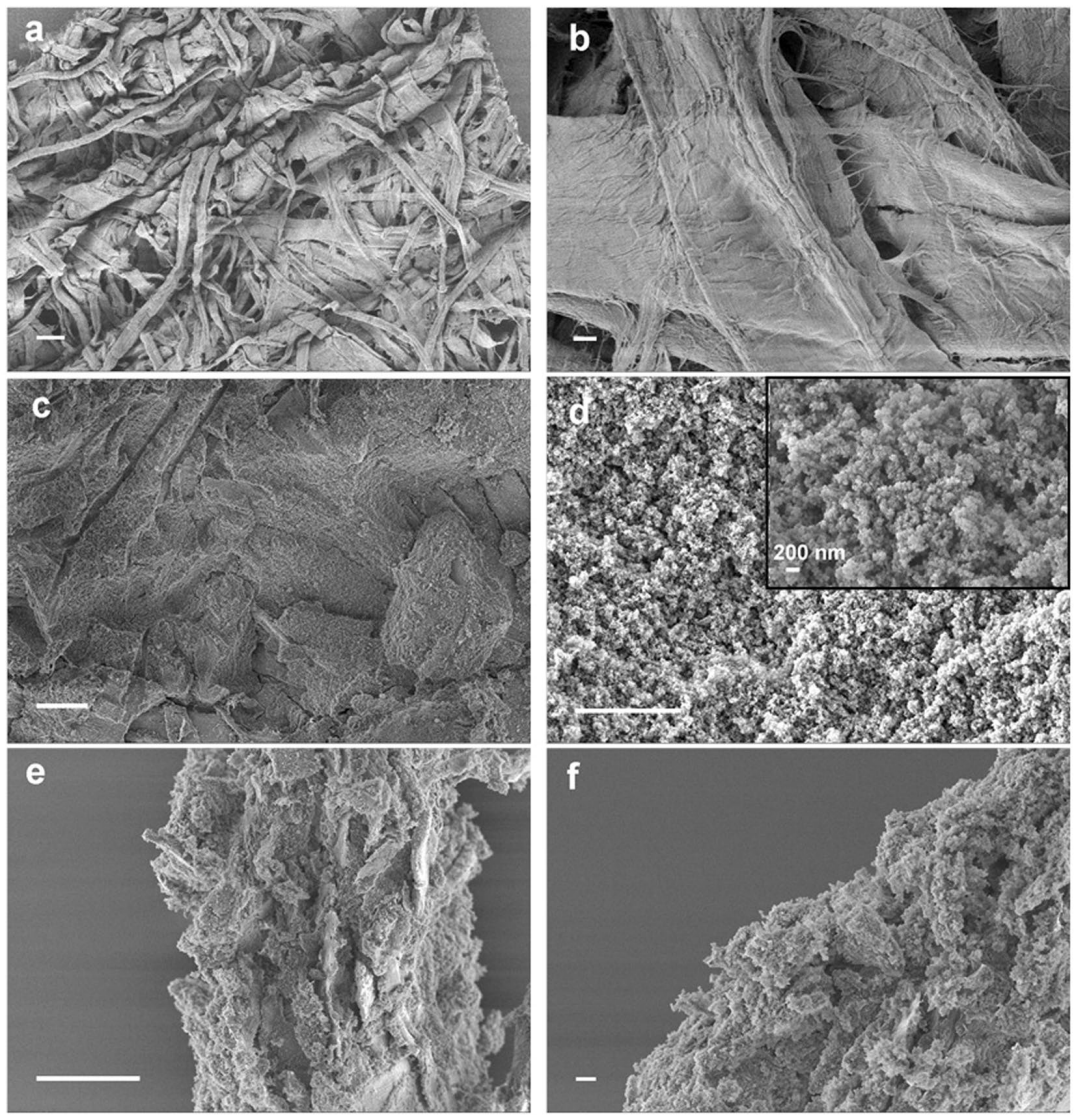
SEM images of the overview and close view of (**a**,**b**) native tissue paper, (**c**,**d**) the front view of porous membrane PPHM-3, and (**e**,**f**) the cross-section of PPHM-3. The scale bar is 50 µm in (**a**,**c**, and **e**) and 5 µm in (**b**,**d** and **f**). The inset is an enlarged view of (**d**).

Scanning electron microscopy (SEM) was used to characterize the morphological evolution of the tissue paper before and after the impregnation process. Figure 2a and b are the SEM images of the native tissue paper. As it can be seen, the tissue paper is a random packing of cellulose microfibers. These fibers are hundreds of µm in length and 50–100 µm in diameter. In comparison, take PPHM-3 as an example, its surface overview in Fig. 2c appears more homogeneous on a micron scale with the cellulose microfibers barely visible, as the copolymer network coats their surface and fills in the interstice among these fibers. A zoom-in view in Fig. 2d however visualized fine textures on a nanoscale, which are in fact the dense aggregates of nanoparticles of 20–30 nm in size. These nanoparticles are the poly(EVImBr-*co*-DVB) copolymers formed during the solvothermal process *via* a nucleation and phase-separation mechanism, as reported previously^46^. Similar nanoparticle morphology was also observed in the cross-sectional view of PPHM-3 (Fig. 2e and f), thus the impregnation occurs in the entire tissue paper interior.

**Figure 3:**
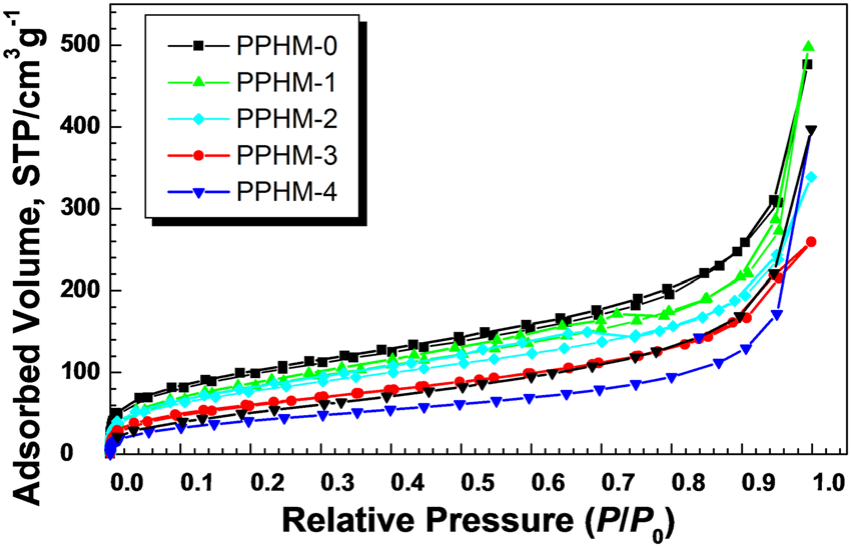
The nitrogen adsorption–desorption isotherms of the PPHMs.

In the absence of any tissue paper, the solvothermal copolymerization of EVImBr and DVB has been reported to produce microporous polymer materials with specific surface area (*S*_BET_, determined by Brunauer–Emmette–Teller equation) controllable in terms of the EVImBr/DVB molar ratio^46^. Thus, PPHMs are subjected to nitrogen gas sorption measurements at 77 K. The sorption isotherms are presented in Fig. 3. Generally speaking, these isotherms are similar to each other, being smooth till *P*/*P*_0_ = 0.85. After this value, a rapid increase in the nitrogen uptake occurs and is associated with the meso- and macropore in the membrane samples. The initial gas sorption below *P*/*P*_0_ < 0.05 is typical for filling of micropores. These micropores stem from the solvothermal copolymerization of the EVImBr and DVB and the micropores are left by solvent evaporation after drying the samples. In this context, the primary nanoparticles observed in Fig. 2d must be microporous themselves. The coexistence of the mesopores is considered to be a nature outcome of the random packing of the nanoparticles, *i.e*. the random interstice among these nanoparticles. This statement is supported by the pore size distribution curve calculated from the non-local density function theory (NLDFT) (Supplementary Fig. S1), in which irregular mesopore size distribution (as these pores are built up from random nanoparticle aggregation.) was observed.

The *S*_BET_ values of PPHMs and the pore volume analysis are summarized in Table 1. The EVImBr-free porous membrane PPHM-0 has a *S*_BET_ of 460 m^2^ g^−1^, being the largest among all PPHMs. The *S*_BET_ values of the other samples decrease upon the increasing addition of EVImBr into the membranes, being 400, 370, 290, and 220 m^2^ g^−1^ for PPHM-x (x = 1 to 4), respectively. Correspondingly the overall pore volume and the pore volume contributed from the micropore portion decrease as well in the same sequence. From the *V*_micro_/*V*_total_ ratios, we can see that below a molar ratio of EVImBr/DVB - 0.2, the micropores contribute 20–31% of the total volume, and the membranes are rich in both micro- and mesopores, while above this ratio, *i.e*. 0.3 in PPHM-4, the micropore volume drops to only 6%, *i.e*. PPHM-4 is practically a mesoporous membrane.

**Table 1:** Porosity properties of PPHMs prepared at different EVImBr/DVB molar ratios.

Entry	PPHM-0	PPHM-1	PPHM-2	PPHM-3	PPHM-4
*S*_BET_ (m^2^ g^−1^)	460	400	370	290	220
*V*_total_ (cm^3^ g^−1^)	0.35	0.27	0.27	0.23	0.24
*V*_micro_(cm^3^ g^−1^)	0.089	0.085	0.075	0.045	0.015
*V*_micro_/*V*_total_	25%	31%	27%	20%	6%

Supplementary Table S2 displays the elemental analysis of the tissue paper and PPHM-3. Based on these data, it is calculated that there is 12.5 wt.% of EVImBr, 40.1 wt.% of DVB, and 47.4 wt.% of tissue paper in the membrane PPHM-3. The total copolymer fraction occupies 52.6 wt.% of PPHM-3. Considering that the tissue paper-free copolymer network prepared under the same conditions has a *S*_BET_ of 570 m^2^ g^−1^, the *S*_BET_ of PPHM-3 is thus proportional to the weight fraction of the copolymers in PPHM-3. That is to say, the surface area of PPHMs comes exclusively from the microporous copolymers impregnated inside. The tissue paper serves simply as the scaffold and does not contribute to the *S*_BET_.

### Switchable oil/water separation

**Figure 4:**
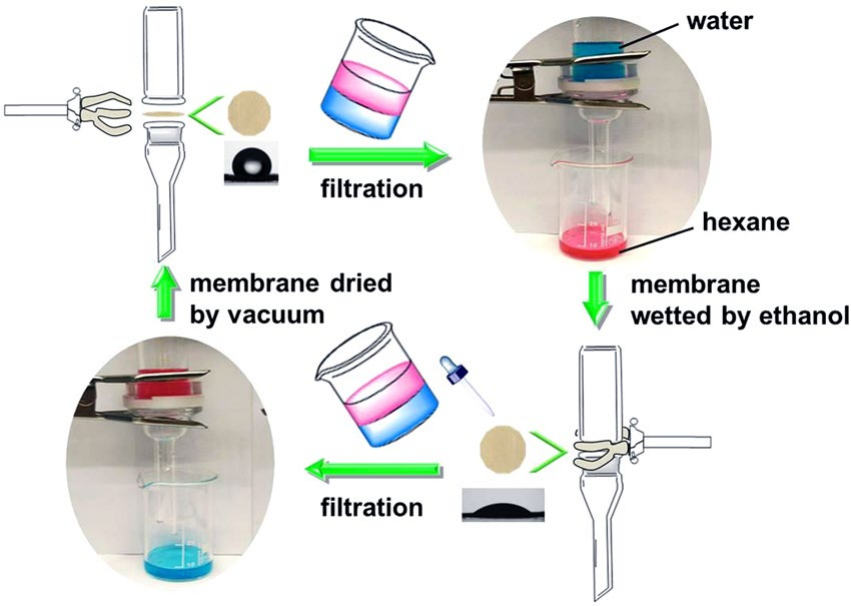
Illustration of the switchable oil/water separation process with the solvent-responsive membrane PPHM-3. The membrane PPHM-3 without treated with ethanol can let hexane pass through but not water; after ethanol treatment, it can let water pass through but not hexane.

The as-synthesized PPHMs were then tested in an oil/water separation experiment shown in Fig. 4. The setup was made of two face-to-face placed Teflon flanges (25 mm in diameter) attached with glass tubes. The porous hybrid membrane PPHM-3 was first examined by this experiment. It was cut round to fit the Teflon flanges and mechanically fixed between them. When pouring a mixture of 10 mL of hexane dyed by Sudan red 7B and 10 mL of water dyed by methylene blue into the separator, the hexane solution flowed through the membrane, leaving the blue aqueous phase in the upper glass tube (see Supplementary Video S1). The mixture of hexane/water was thus successfully separated into two individual homogeneous solutions in hexane and in water, respectively, by the porous hybrid membrane.

The surface property of PPHM-3 was then analysed by static water contact angle (WCA) measurement, which was carried out in atmospheric environment (25 °C, 50% RH). Dropping water onto the surface of the membrane PPHM-3 forms a stable droplet with a WCA of 118 ± 2° (Supplementary Fig. S2), proving the native surface is indeed hydrophobic. It naturally blocks the wetting of membrane by water. By contrast, when a hexane droplet touches the surface, it is immediately absorbed due to the strong affinity towards the oleophylic membrane surface and then diffusion into the membrane by capillary force (Supplementary Fig. S3). These two experiments explained the selective filtration of hexane solution from a hexane/water mixture by PPHM-3. It should be noted that the native tissue paper is intrinsically hydrophilic due to the hydroxyl rich cellulose surface. It can nevertheless pass either water or hexane through, because the pores in the native tissue paper are tens of microns in size, thus the surface property is not dominant in controlling the filtration process.

**Figure 5:**
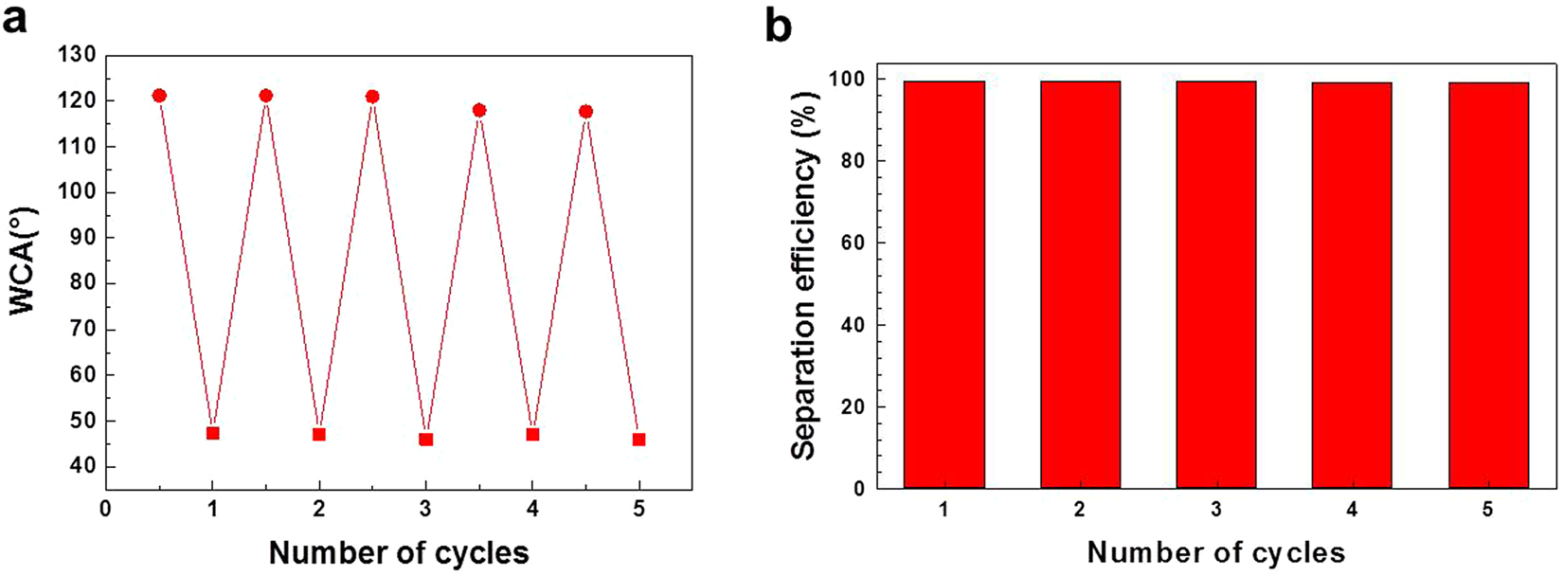
(**a**) Reversible wettability of the membrane PPHM-3 for water droplets. The membrane was repeatedly treated by ethanol and dried in vacuum oven at 80 °C for 12 h. (**b**) The separation efficiency of PPHM-3 at different cycles.

After the oil/water separation experiment, the membrane PPHM-3 was thoroughly wetted by ethanol and then dried in air in fume hood. It was then restored into the setup. When the same mixture of water and hexane solution was poured into the separator, the blue phase, *i.e*. aqueous solution can pass through while hexane cannot (see Supplementary Video S2). In the WCA measurement of the ethanol-treated membrane, water was found to diffuse through the membrane (Supplementary Fig. S4), *i.e*. a hydrophilic surface. Assisted by digital camera, a WCA of 47 ± 2° was determined for water on the membrane surface. The ethanol effect can be observed as long as its ethanol content is no less than 0.8 wt.%. This value is so low that once the membrane is wetted by ethanol, it keeps its surface affinity towards water, unless ethanol was forced to be completely removed under simultaneous vacuum and thermal treatment, here 10^−2^ mbar and 80 °C for 12 h. After this treatment, the membrane recovers its capability to filter hexane solution but block aqueous solution. The reversible operation of the membrane PPHM-3 upon ethanol and vacuum treatment can be repeated for five cycles without losing its separation capability and efficiency. The corresponding wettability transition of the membrane PPHM-3 was shown in Fig. 5a. The separation efficiency *η* was evaluated by *η* = (*m*_1_/*m*_0_) × 100, where m_0_ and m_1_ are the mass of the rejected liquid phase before and after the separation process. The separation efficiency of PPHM-3 for each cycle is above 99% as can be seen in Fig. 5b.

### Tuneable property

**Figure 6:**
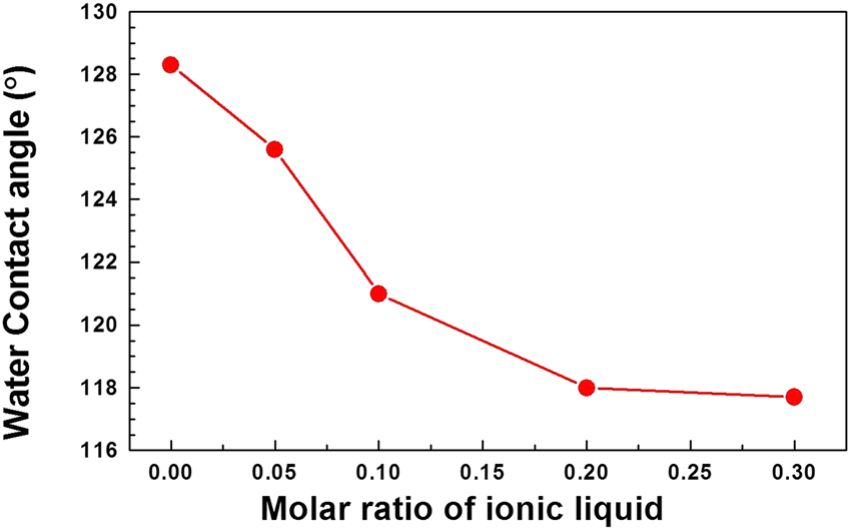
The water contact angles of the porous membranes PPHM-0 (0.0), PPHM-1 (0.05), PPHM-2 (0.1), PPHM-3 (0.2), and PPHM-4 (0.3).

Achieving the success of oil/water separation by PPHM-3 in a repeatable and reversible manner, we expanded our study immediately to the full series of PPHMs with different molar ratios of EVImBr/DVB. The water contact angle measurements of the membranes were showed in Fig. 6. All of them are hydrophobic, WCA being larger than 117°. A clear trend is that with the increase in the content of ionic liquid in the copolymer membranes, the water contact angles of the PPHMs drops from 128° till a plateau at 117° (Fig. 6 and Supplementary Table S3). This is reasonable, since EVImBr is hydrophilic. The more EVImBr is incorporated, the less hydrophobic the membrane surface is.

These PPHMs were further tested in the switchable oil and water separation experiments. The results are collected in Supplementary Table S4. All of these PPHMs before ethanol treatment could filter hexane solution and repel the entrance of water. After ethanol treatment, the PPHMs displayed different separation performances. All membranes with the EVImBr/DVB molar ratio lower than 0.1 cannot be used to filter aqueous solution, as due to too less ionic liquid content they are too hydrophobic even after ethanol treatment. By contrast, all membranes with EVImBr/DVB of 0.1 or above serve efficiently for switchable oil/water separation. It seems indeed that the ionic liquid fraction plays a key role in the switchable separation process, as reported in previous literature that solvent could modulate the surface chemical structure^23–26^. We further studied the membrane PPHM-3 (Br^−^) by anion exchange with large-sized fluorinated counteranions such as BF_4_^−^, PF_6_^−^, and TFSI^−^. All of the anion-exchanged membranes after ethanol treatment lost the filtration selectivity, and both hexane and water passed through them freely. The results are summarized in Supplementary Table S5. These experiments indicate that the switchable oil/water separation function is ion-specific, *i.e*. only Br^−^ containing PPHMs can serve as “smart” hybrid membranes for switchable oil/water separation.

## Discussion

**Figure 7:**
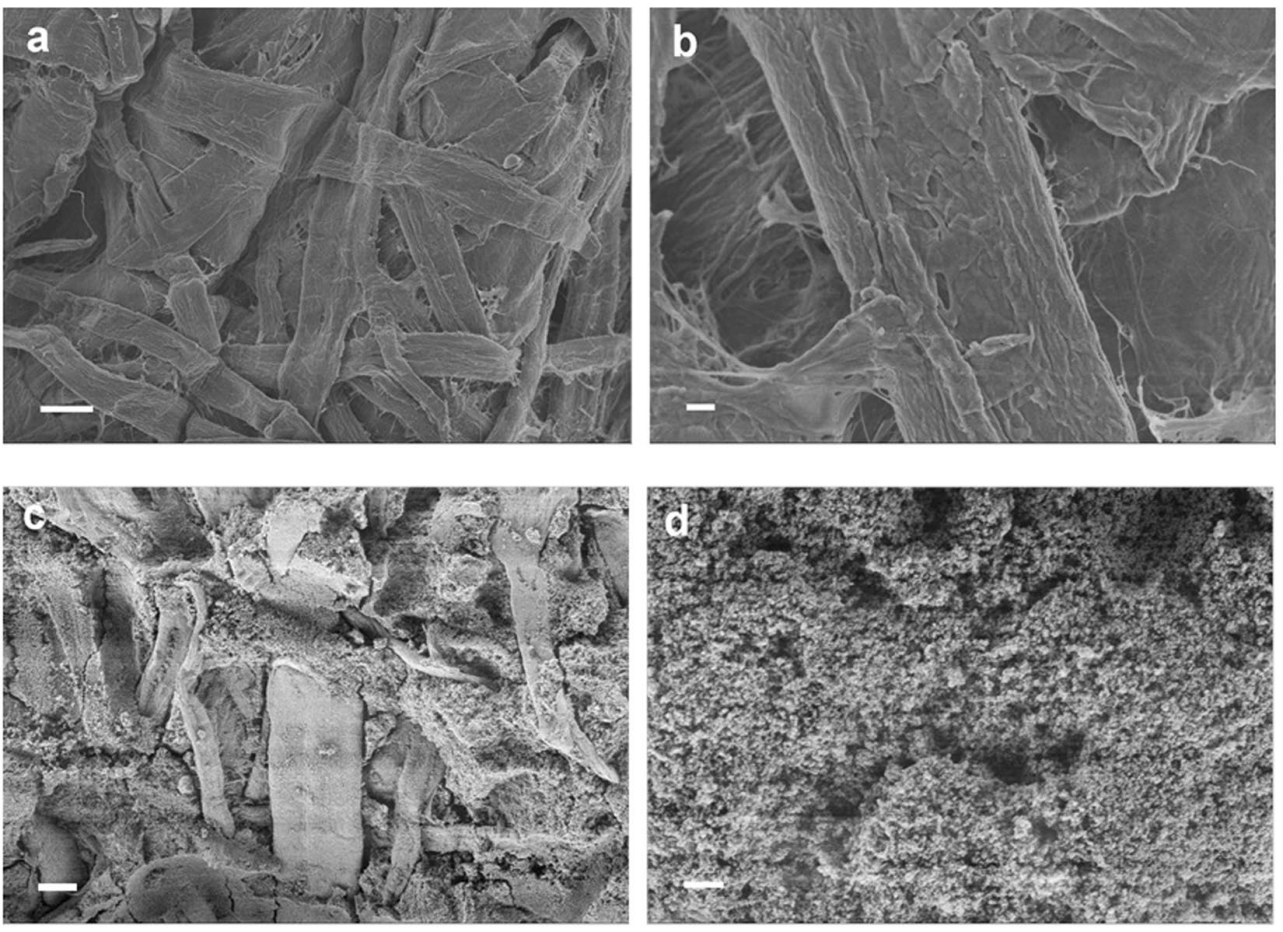
SEM images of the porous membrane PPHM-3 (**a**,**b**) after ethanol treatment and (**c**,**d**) after drying under vacuum oven at 80 °C for 12 h. The scale bar is 20 µm in (**a**,**c**) and 2 µm in (**b**,**d**).

To reveal the mechanism of the switchable oil/water separation behaviour, we applied SEM characterization of the PPHMs after ethanol treatment. The native PPHM-3 is coated with dense nanoscale aggregates on their surface (Fig. 2d) that create a submicron-scale roughness of the surface. According to the Wenzel law^54^, surface roughness will amplify but not switch the surface hydrophilicity/hydrophobicity, from which it is deduced that the surface at this stage is intrinsically hydrophobic. After ethanol treatment and drying thoroughly at room temperature, the surface of the membrane PPHM-3 was visualized by SEM, which showed a surprisingly smoother surface (Fig. 7a and b). The water contact angle measurement shows a lower WCA of 47 ± 2° (Fig. 6), i.e. a hydrophilic surface. When the membrane was fully dried in vacuum oven at 80 °C for 12 h, the surface morphology profile and the water contact angle of the membrane PPHM-3 are fully reverted to the original state (Fig. 7c and d).

These experiments implies that the surface structure of the membrane PPHM-3 has changed after ethanol wetting and it can recover its original state by applying vacuum and thermal treatment simultaneously due to the entire removal of ethanol. The change of the surface topography observed in the SEM characterization is indicative of the change of the chemical structure dominating the topmost surface due to different responses of the hydrophobic DVB and the hydrophilic EVImBr domains in the copolymer to solvent. The membrane freshly prepared by solvothermal copolymerization is hydrophobic, as confirmed by the WCA measurements. The fraction of the EVImBr ionic liquid units in the poly(EVImBr-*co*-DVB) network can decrease the hydrophobicity in a small range, as indicated by decreased WCA values from 128° to 117° when increasing the ionic liquid content (Fig. 6). It should be noted that these values are far below the superhydrophobic regime, indicating a relatively weak hydrophobicity (but still hydrophobic enough to block water entrance) caused by the surface coating of the DVB fraction on the hydrophilic cellulose fibres. After wetted by ethanol, the EVImBr units interact very strong with ethanol through the well-known polarization and hydrogen bonding^55–60^, thus the EVImBr domains in the poly(EVImBr-*co*-DVB) network are preferentially oriented outside to face the solvent, switching the surface from being weakly hydrophobic to being hydrophilic. This hypothesis is supported by the anion exchange experiment, which changed the hydrophilic EVImBr units into hydrophobic ones thus lost their function to modulate the surface hydrophobicity/hydrophilicity.

In summary, a facile solvothermal copolymerization route was developed to prepare porous polymer-paper hybrid membranes. The function of the tissue paper is to serve as skeleton and scaffold to accommodate the later-introduced porous poly(IL-co-DVB) copolymer network built up during the solvothermal process. The solvent-responsive surface chemical structure of the membranes leads to the intriguing switchable wettability, which enables controllable oil/water separation. The ionic liquid fraction in the copolymer is a key factor to the switchable separation. The rather simple fabrication process and scalable production are extra merits of this approach which may facilitate their real life applications.

## Methods

### Materials

Divinylbenzene (DVB, 80% mixture of isomers), *N*-vinyl imidazole (VIm, 99%), sodium tetrafluoroborate (NaBF_4_, 98%), *N,N*-dimethylformamide (99.8%), methylene blue (98%), and Sudan red 7B (98%) were obtained from Sigma-Aldrich. 1-Bromoethane (98%), and potassium hexafluorophosphate (KPF_6_, 99%) were obtained from Alfa Aesar. Lithium bis(trifluoromethane sulfonyl)imide (LiTFSI, 99%) was obtained from IOLITEC Ionic liquids Technologies GmbH. *n*-Hexane was obtained from VWR International. 2,2′-Azobis(2-methylpropionitrile) (AIBN, 98%, Sigma-Aldrich) was recrystallized from methanol before use. The ionic liquid monomer 1-ethyl-3-vinylimidazolium bromide (EVImBr) was synthesized according to previous work^46^. Water used in the experiments was deionized. The tissue paper was purchased from Kimberly-Clark.

### Instrumental characterization

Field emission scanning electron microscopy (FESEM) was performed on a LEO 1550-Gemini instrument at an accelerating voltage of 0.5–6.0 kV. The samples on carbon coated stubs were coated by sputtering an Au-Pd alloy prior to examination. Nitrogen sorption measurements were performed on a Quantachrome Autosorb-1 at 77 K and data analysis was performed by Quantachrome software. Samples were degassed at 80 °C for 20 h prior to measurements. The specific surface area was calculated using the Brunauer-Emmette-Teller (BET) equation and the pore size distribution was calculated from the non-local density functional theory (NLDFT) method. The contact angle measurements were conducted using a Kruss G10 contact angle measurement system by dropping a small volume of liquid (~2 µL) from a 2-mL micrometer syringe (Gilmont) onto the target surface. At least three measurements were performed on each sample. The typical error in measurements was ±2°. Elemental analysis was performed for carbon, hydrogen, nitrogen and sulfur using a Vario EL Elemental Analyzer. The water contents in the collected filtrates were determined by Mettler Toledo C20 Coulometric Karl Fischer Titrator.

### Preparation of PPHMs

The synthetic route was conducted as follows. A defined amount of the monomeric ionic liquid EVImBr and 0.1 g of AIBN were loaded into a 20 mL DMF solution of DVB (4.0 g). The mixture was stirred at room temperature for 3 h before it was transferred into a Teflon-sealed autoclave containing a roll of five sheets of tissue paper and then solvothermally treated at 180 °C for 24 h. After cooling down, the PPHMs were isolated from the entire monolith and immersed in DMF to remove any unreacted monomer for 24 h. The PPHMs were dried in a fume hood to slowly evaporate the residual solvent. After one day it was further dried in a high vacuum oven at 80 °C for 24 h.

### Ion exchange of PPHMs

To exchange the anion in the porous network from Br^−^ to tetrafluoroborate (BF_4_^−^), a sheet of 20 × 10 cm^2^ PPHM-3 was immersed in a bottle containing a solution of 80 mL water and 654 mg NaBF_4_. The molar ratio of the ionic liquid species in PPHM-3 and NaBF_4_ is about 1:10 to ensure a thorough anion exchange process. The bottle was put on a shaker at a vibration rate of 200 rpm for 24 h and then the membrane was rinsed with water several times before dried at 80 °C for 24 h under high vacuum (10^−2^ mbar). The same procedure was employed for exchanging the anion to hexafluorophosphate (PF_6_^−^) and bis(trifluoromethane sulfonyl)imide (TFSI^−^).

## Electronic supplementary material

Supplementary video S1

Supplementary video S2

Supplementary Information
