# Supplementary material for: Synthesis of porous polymer/tissue paper hybrid membranes for switchable oil/water separation
Source: Sci Rep. 2017 Jun 8;7:3101. doi: 10.1038/s41598-017-03265-z (PMC5465062; doi:10.1038/s41598-017-03265-z)
Supplement: Supplementary file 3 — Supplementary Information [file 41598_2017_3265_MOESM3_ESM.pdf]

Supplementary Information for

Synthesis of porous polymer/tissue paper hybrid membranes for switchable oil/water separation

Cong-Xiao Cao,[a,b,c] Jia-Yin Yuan,*[,c,d] Jin-Pei Cheng,[a] Bao-Hang Han*[,b]

[a] *State Key Laboratory of Elemento-organic Chemistry, Collaborative Innovation Center of Chemical Science and Engineering (Tianjin), College of Chemistry, Nankai University, Tianjin 300071, China*

[b] *CAS Key Laboratory of Nanosystem and Hierarchical Fabrication, CAS Center for Excellence in Nanoscience, National Center for Nanoscience and Technology, Beijing 100190, China*

[c] *Department of Colloid Chemistry, Max Planck Institute of Colloids and Interfaces, Potsdam D-14424, Germany*

[d] *Department of Chemistry and Biomolecular Science, and Center for Advanced Materials Processing, Clarkson University, Potsdam, New York 13699-5814, USA.*

Tel.: +86 10 8254 5576. Email: hanbh@nanoctr.cn

Tel.: +1 315 268 4247. Email: jyuan@clarkson.edu

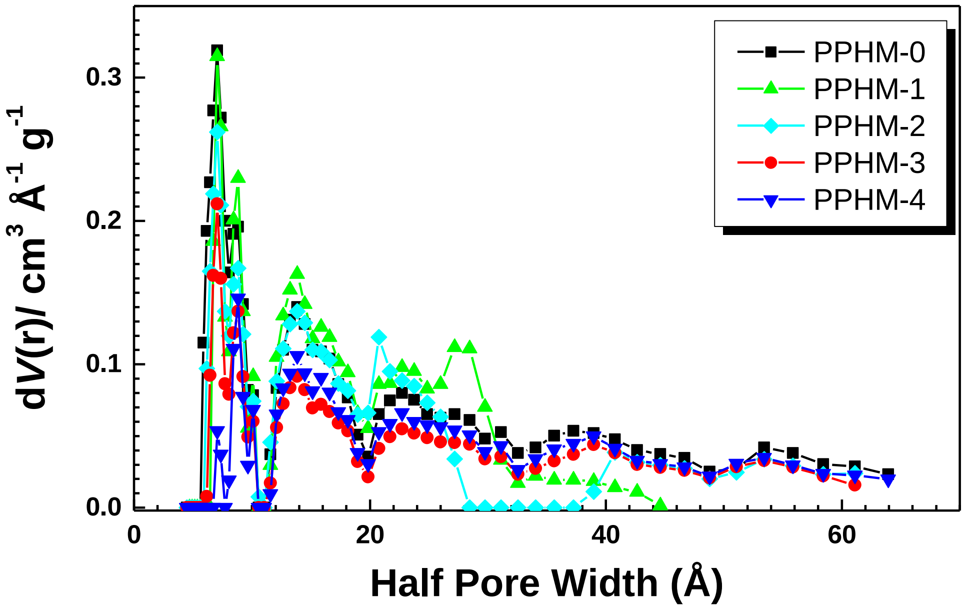

Supplementary Figure S1 The pore size distribution profiles of the PPHMs.

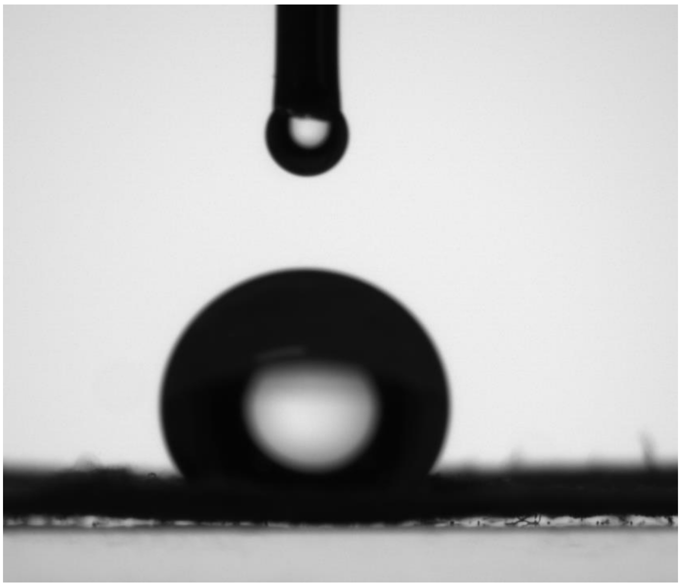

Supplementary Figure S2 The image of the water droplet on the membrane PPHM-3.

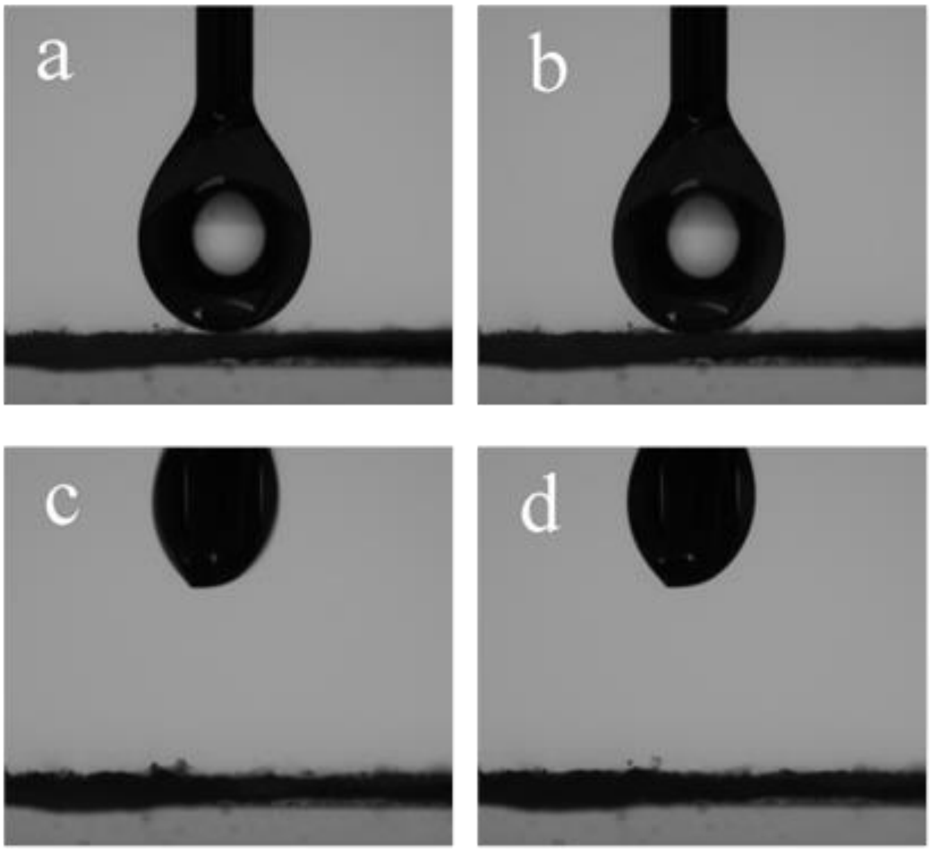

Supplementary Figure S3. The photographs of the hexane droplet on the membrane PPHM-3 surface in air at 0.1 s (a), 0.2 s (b), 0.3 s (c), and 0.4 s (d).

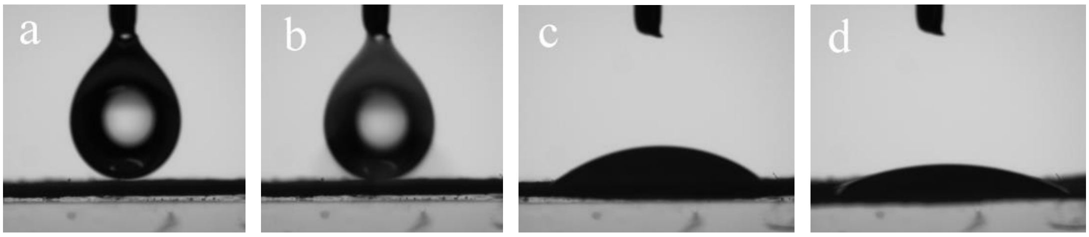

Supplementary Figure S4 The images of the water droplet on the solvent-treated membrane PPHM-3 surface in air at 0.1 s (a), 0.2 s (b), 0.3 s (c), and 2.2 s (d).

Supplementary Table S1 Synthetic conditions of PPHMs

Entry	DVB (g)	EVImBr (g)	AIBN (g)	DMF (mL)	Temperature (℃)	Time (h)	Tissue paper (sheets)
PPHM-0	4.0	0	0.1	20	180	24	5
PPHM-1	4.0	0.31	0.1	20	180	24	5
PPHM-2	4.0	0.62	0.1	20	180	24	5
PPHM-3	4.0	1.24	0.1	20	180	24	5
PPHM-4	4.0	1.86	0.1	20	180	24	5

Supplementary Table S2. Elemental analysis of tissue paper and PPHM-3

Sample	N (%)	C (%)	H (%)	S (%)
Tissue Paper	0.19	41.8	6.13	0.754
PPHM-3	1.82	57.0	6.57	0.712

Supplementary Table S3 Water contact angle of PPHMs

Entry	PPHM-4	PPHM-3	PPHM-2	PPHM-1	PPHM-0
WCA (°)	117.7±2	118.0±2	121.0±2	125.6±2	128.3±2

Supplementary Table S4 The performance of the polymer-paper hybrid membranes with different EVImBr/DVB molar ratios for solvent-responsive switchable oil/water separation

	Before treatment		After treatment	
	n-Hexane	Water	n-Hexane	Water
Tissue Paper	√	√	√	√
PPHM-0	√	×	√	√
PPHM-1	√	×	√	√
PPHM-2	√	×	×	√
PPHM-3	√	×	×	√
PPHM-4	√	×	×	√

Supplementary Table S5 The performance of the polymer-paper hybrid membranes with different counteranions for solvent-responsive switchable oil/water separation

	Before treatment		After treatment	
	n-Hexane	Water	n-Hexane	Water
PPHM-3-Br	√	×	×	√
PPHM-3-BF_4	√	×	√	√
PPHM-3-PF_6	√	×	√	√
PPHM-3-TFSI	√	×	√	√
